# Long-term survival outcomes following cytoreductive surgery and hyperthermic intraperitoneal chemotherapy for peritoneal metastasis of hepatocellular carcinoma patients

**DOI:** 10.1186/s12957-024-03426-1

**Published:** 2024-05-31

**Authors:** Guojun Yan, Kai Zhang, Lijun Yan, Yanbin Zhang

**Affiliations:** Department of Peritoneal Cancer Surgery, Beijing Shijitan Hospital, Capital Medical University, No. 10, Tieyi Road, Yangfangdian Street, Haidian District, Beijing, 100038 China

**Keywords:** Cytoreductive surgery, Hyperthermic intraperitoneal chemotherapy, Peritoneal metastasis, Hepatocellular carcinoma

## Abstract

**Background:**

Hepatocellular carcinoma with peritoneal metastasis (HCC-PM) has a poor outlook. Traditional treatments have limited effect on survival. The safety and efficacy of cytoreductive surgery with hyperthermic intraperitoneal chemotherapy (CRS + HIPEC) have been shown in other peritoneal cancers. This study evaluates the role of CRS + HIPEC in HCC-PM.

**Methods:**

A retrospective analysis of HCC-PM patients treated with CRS + HIPEC at Beijing Shijitan Hospital from March 2017 to December 2023 was conducted, assessing clinical features, severe adverse events (SAEs), and overall survival (OS) rates.

**Results:**

The study population comprised 10 HCC-PM patients who underwent CRS + HIPEC. The median peritoneal cancer index (PCI) was 25, and complete cytoreduction (CC0 ~ 1) was achieved in half of the patients. Three patients experienced SAEs within 30 days postoperatively. The 1-year, 3-year, and 5-year OS rates were recorded as 89.0%, 89.0%, and 21.0% respectively, with a median OS1 of 107.8 months and OS2 of 49.9 months. The median progression-free survival (PFS) was 5.0 months.

**Conclusion:**

The application of CRS + HIPEC offers significant benefits to patients with HCC-PM. A selected group of patients may achieve prolonged PFS. Incorporating CRS + HIPEC into the treatment paradigm can thus be considered a strategic therapeutic option for patients with HCC-PM.

## Introduction

The incidence of malignant tumors is on the rise globally, presenting a significant challenge to healthcare systems worldwide. Recent global cancer statistics revealed that hepatocellular carcinoma has ascended to become the sixth most common malignancy and the third leading cause of cancer-related fatalities internationally. Notably, China bears a disproportionate share of the burden, accounting for approximately 45.27% of new cases and 47.12% of deaths globally [1]. The occurrence of extrahepatic metastasis in hepatocellular carcinoma treatment represents a particularly formidable obstacle, hindering the effectiveness of various therapeutic strategies including chemotherapy, targeted therapy, and immunotherapy, which have shown limited success in enhancing patient survival rates. Hepatocellular carcinoma with peritoneal metastasis (HCC-PM) is a relatively uncommon condition, with an incidence rate ranging between 2.0 and 18.0% [2, 3], and a median overall survival (OS) time spanning from 6.0 to 14.0 months [4, 5].

The combination of cytoreductive surgery and hyperthermic chemotherapy (CRS + HIPEC) has established its safety and efficacy in the treatment of an array of cancer types, including pseudomyxoma pertonei, mesothelioma, ovarian cancer, as well as selected cases of peritoneal metastases from colorectal and gastric cancer [6–10]. Furthermore, an increasing body of research has illuminated the potential advantages of CRS + HIPEC in managing HCC-PM, demonstrating that a highly selective cohort of patients can achieve prolonged survival [2, 3, 11–17]. Nevertheless, the precise application and therapeutic efficacy of this integrated approach in treating HCC-PM remain areas of active investigation and some uncertainty.

In response to these clinical concerns and to further elucidate the role of CRS + HIPEC in HCC-PM management, we conducted a retrospective study to critically evaluate the safety and efficacy associated with this treatment modality. Our objective is to contribute to the ongoing dialogue regarding optimal therapeutic strategies for HCC-PM, with the ultimate goal of improving patient outcomes.

## Methods

We conducted a retrospective analysis of clinical data from patients diagnosed with HCC-PM who underwent CRS + HIPEC at the Department of Peritoneal Cancer, Beijing Shijitan Hospital, between March 2017 and December 2023. Cases of synchronous peritoneal metastasis were identified by simultaneous detection of hepatocellular carcinoma and peritoneal metastasis, whereas metachronous peritoneal metastasis was defined as peritoneal metastasis occurring post-hepatic resection. The research methodology adhered strictly to the ethical guidelines set forth by the institutional research committee and aligned with the principles of the 1964 Helsinki Declaration and its subsequent amendments. The study was approved by the Ehics Committee of Beijing Shijitan Hopsital. Written informed consent was obtained from all patients who underwent CRS + HIPEC after a thorough explanation of the procedure and its potential risks.

The decision to proceed with CRS + HIPEC for HCC-PM was based on a set of preoperative inclusion criteria designed to identify patients who might benefit from this aggressive treatment approach, despite it not being the standard of care for this patient population. The criteria included: (1) Diagnosis of HCC with biopsy-confirmed or frozen pathology-verified peritoneal metastasis. (2) Evaluation of the extent of disease through imaging studies, such as CT scans or PET scans, indicating that the peritoneal metastases were deemed potentially resectable. (3) Assessment of the patient’s overall health status and performance status, ensureing they were fit enough to endure major surgery and the associated risks. (4) Adequate hepatic reserve and function, as determined by laboratory tests, to withstand the potential impact of the procedure on the live. (5) Multidisciplinary team consensus, involving hepatobiliary surgeons, gastrointestinal surgeons, medical oncologists, radiologists, and pathologists, that the patient would likely benefit from the CRS + HIPEC approach. (6) Informed consent from the patient, including a thorough discussion of the risks, benefits, and alternative treatment options.

The extent of disease spread was evaluated using the intraoperative peritoneal cancer index (PCI), which involves dividing the abdominal cavity into 13 zones and scoring the diameter of the largest tumor nodule in each zone according to the PCI scoring system: 0 indicates no visible nodule, 1 for a nodule ≤ 0.5 cm in diameter, 2 for a nodule between 0.5 cm and 5.0 cm, and 3 for a nodule > 5.0 cm or fused into a piece, with scores ranging from 0 to 39. Following CRS, the completeness of cytoreduction (CC) score was assessed: CC0 indicated no visible nodule; CC1 indicated residual nodules < 2.5 mm in diameter; CC2 indicated residual nodules between 2.5 mm and 2.5 cm; CC3 indicated residual nodules > 2.5 cm in diameter or fusion [18].HIPEC was performed using cisplatin 120 mg and docetaxel 120 mg, dissolved in saline, and administered at temperatures between 42 ℃ and 43 ℃ for 60 min. An open technique was used for even distribution of the chemotherapy agents, and temperature probes monitored the intraperitoneal heat without causing systemic hypermia. After HIPEC, digestive tract reconstruction was carried out, followed by closure of the abdomen [19].Details of the CRS + HIPEC procedure, including operation time, organ resections, peritoneal resections, anastomoses, HIPEC, PCI score, CC score, blood transfusion requirements, and ascites, were meticulously analyzed. Organ resections encompassed procedures on the ascending colon, transverse colon, descending colon, sigmoid colon, total colon, gastrectomy, small intestine resection, rectal resection, ovarian and fallopian tube resection, hysterectomy, partial hysterectomy, kidney and spleen resection, pancreas, gallbladder resection, and bladder resection. Peritonectomy involved bilateral diaphragmatic peritoneum, greater and lesser omentum, bilateral colonic sulcus peritoneum, hepatic round ligament, anterior wall peritoneum, pelvic floor peritoneum, and mesentery. Postoperative complications were graded using the Clavien-Dindo Classification System, which categorizes 48 adverse events into nine levels, with levels III to IV defined as severe adverse events (SAEs) [20].

Patient surveillance involved outpatient visits or phone consultations, including physical examinations, blood tests, alpha-fetoprotein levels, and abdominal and chest CT scans every three months during the first year, followed by every six months thereafter. For patients who underwent multiple CRS + HIPEC treatments, survival analyses commenced from the date of their first procedure at our center. OS was categorized into two types: OS1, which represents the time from HCC diagnosis to death or last follow-up, and OS2, defined as the time from CRS + HIPEC to death or last follow-up. Progression-free survival (PFS) was measured from the time of CRS + HIPEC until tumor progression or recurrence was detected. The final day for our analysis was December 31, 2023.

Statistical analysis was performed using SPSS 26.0 software (IBM Corp., Armonk, NY). The Kaplan-Meier and Life Table methods were utilized to estimated OS and PFS, with comparisons made using the log-rank text. Correlation analysis was conductied using the Pearson correlation coefficient. Prognostic factors were analyzed using logistic regression.

## Results

In this study, a total of 10 patients were enrolled, comprising 3 synchronous and 7 metachronous HCC-PM cases. Following hepatic resection, all metachronous HCC-PM patients received subsequent treatments, including sorafenib, chemotherapy, regorafenib, PD-1 blockade, ablation, and transcatheter arterial chemoembolization (TACE). The characteristics of the 10 patients are summarized in Table 1.

**Table 1:** Characteristics of 10 patients who underwent CRS + HIPEC

Variables	Gender	Age	BMI	KPS	Syn/Met	Treatment before CRS + HIPEC	Pathology
1	Male	38	22.6	90	Syn	Diagnostic laparoscopy; Sorafenib	HCC
2	Male	43	22.5	90	Met	HR; TACE; Surgery; LBP + LV; Sorafenib; Levatinib	HCC
3	Male	54	24.8	100	Met	HR; Surgery; Tegafur + Gemcitabine	HCC
4	Male	32	26.9	100	Met	HR; Surgery; PD-1 blockade; LOHP + CAP + PD-1 blockade + Regorafenib	HCC
5	Female	66	20.0	80	Syn	No	No
6	Female	48	24.3	90	Met	Ablation; TACE; Sorafenib	HCC
7	Male	42	19.5	90	Met	TACE; OLT; Sorafenib; Lenvatinib; Srugery; mFOLFOX6 + Bevacizumab	HCC
8	Male	54	31.3	90	Met	PD-1 blockade; Sorafenib; Lenvatinib; Nimotuzumab; Regorafenib; Radioactive seed implantation	HCC
9	Male	41	21.6	90	Met	TACE; Sorafenib; Surgery	HCC
10	Female	44	21.9	90	Syn	No	No

Met: metachronous; Syn: synchronous; HR: hepatic resection; TACE: transcatheter arterial chemoembolization; LBP: lobaplatin; LV: levofolinate; OLT: liver transplantation; LOHP: oxaliplatin; CAP: capecitabine; HCC: hepatocellular carcinoma; mFOLFOX6: oxaliplatin + 5-Fu + levofolinate

**Fig. 1:**
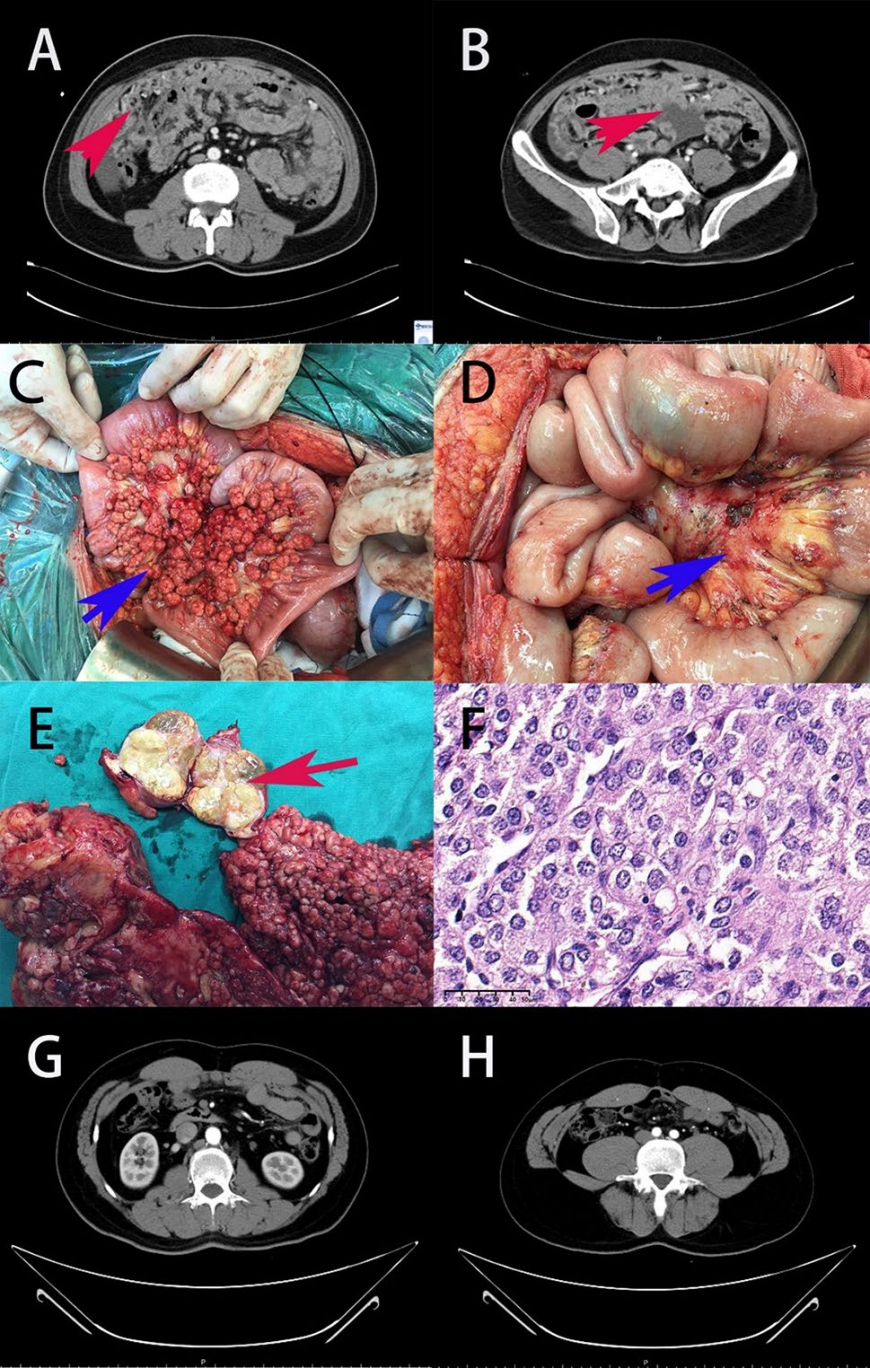
**A**, **B**: Preoperative abdominal CT scans revealed a diffuse distribution of tumors throughout the abdomen, with the formation of an “omental cake.” **C**: Intraoperatively, diffuse nodules were observed within the intestinal mesentery. **D**: All nodules were excised following CRS + HIPEC. **E**: The extent of multiple specimens was displayed and documented. **F**: Pathology depicted hepatocellular carcinoma cells at 40x magnification. **G**, **H**: No signs of recurrence were evident until the latest follow-up, a CT scan almost 7 years post-CRS + HIPEC

The median PCI was 25, with patients having a median age of 43.5-year at the time of CRS + HIPEC, and a median operation time of 509 min. Half of the patients achieved CC0 to CC1 resections. CC0/1 was associated with lower PCI scores (*P* < 0.05). Details of the CRS + HIPEC procedures are summarized in Table 2; Fig. 1.

**Table 2:** Operative data of patients undergoing CRS + HIPEC

Variables	PCI score	CC score	OR time (min)	Organ resections	Peritoneal resections	Ascites (ml)	Anastomosis	HIPEC	Blood loss(ml)	Blood transfusion (U)
1	30	2	785	1	5	2000	0	Yes	6000	14
2	12	2	500	2	6	0	2	Yes	400	0
3	24	3	826	2	4	600	2	Yes	500	0
4	30	2	705	2	7	100	2	Yes	900	2
5	33	2	518	4	9	2000	0	Yes	500	2
6	10	0	415	2	6	0	0	Yes	300	0
7	25	0	679	1	6	0	1	Yes	800	4
8	30	3	440	0	5	1500	0	Yes	200	2
9	9	0	391	3	2	0	2	Yes	500	6
10	6	0	200	3	2	100	0	Yes	100	0

**Table 3:** Postoperative treatment and outcome data

Variables	SAE	Treatment after CRS + HIPEC	PFS	Survival	OS1 (months)	OS2 (months)
1	No	DTX + DDP IP followed by DTX + CBP Ivgtt	82.7	Yes	85.1	82.7
2	No	Repeated surgery	22.1	Yes	153.9	62.6
3	Yes	FOLFORI + Bevacizumab; Bevacizumab; PTX; Gemcitabine	4.0	No	127.7	5.5
4	No	DTX + CBP; DTX + CBP + PD-1 blockade + Levatinib; repeated surgery	19.0	No	54.2	36.9
5	Yes	No	2.1	No	2.1	2.1
6	No	Sorafenib	44.5	No	107.8	49.9
7	No	Chemotherapy + Bevacizumab	3.0	Yes	58.0	26.1
8	Yes	No	1.0	No	55.6	1.0
9	No	No	5.0	Yes	9.0	5.0
10	No	TACE; PD-1 blockade + Sorafenib; repeated surgery	12.7	Yes	43.0	43.0

IP: intraperitoneal chemotherapy; FOLFORI: irinotecan + fluorouracil + levofolinate; TACE: transcatheter arterial chemoembolization; DTX: docetaxel; DDP: cisplatin; CBP: carboplatin; PTX: paclitaxel

**Fig. 2:**
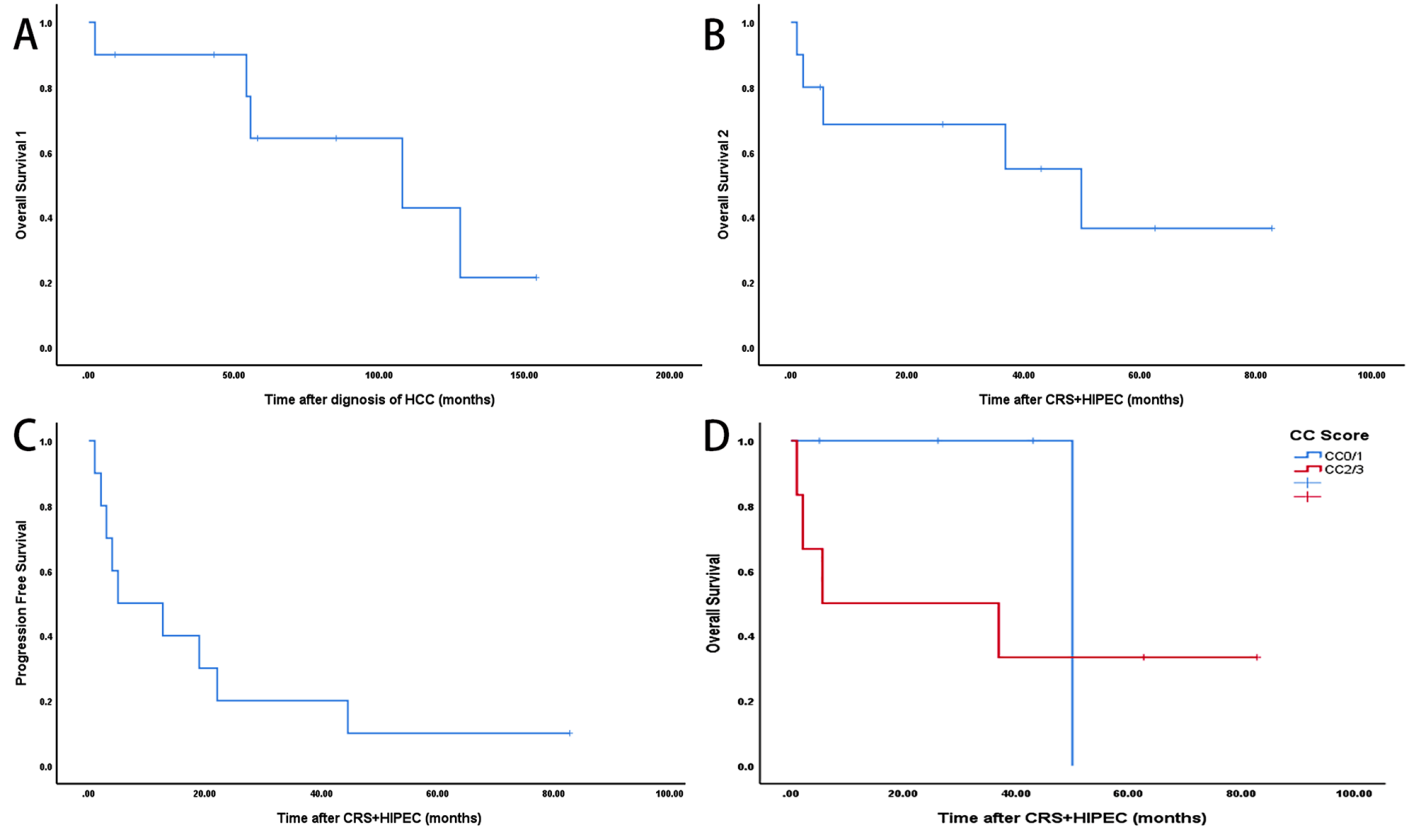
**A** OS1 from the diagnosis of HCC to the last follow-up/death. **B**: OS2 from CRS + HIPEC to the last follow-up/death. **C**: PFS from CRS + HIPEC to the last follow-up/death. **D**: No significant differences in overall survival were observed when comparing patients with HCC-PM in both CC0/1 and CC2/3 groups

However, three patients experienced SAEs, including one case of incisional dehiscence and one case of pleural effusion. Regrettably, one patient died within 30 days postoperatively. Some patients underwent chemotherapy, targeted therapy, and repeat surgery after CRS + HIPEC (Table 3). The median follow-up period was 31.5 months following the initial CRS + HIPEC. The median OS1 was 107.8 months, the median OS2 was 49.9 months, and the median PFS was 5.0 months. The OS rates at 1-year, 3-year, and 5-year after the diagnosis of HCC were 89.0%, 89.0%, and 21.0%, respectively, and after CRS + HIPEC were 68.0%, 53.0%, and 35.0%, respectively. The PFS rates at 1-year, 3-year, and 5-year were 50.0%, 10.0%, and 10.0%, respectively. However, we did not find a significant difference in overall survival based on the CC score alone (CC0/1 versus CC2/3) (*P* > 0.05). The CC score was not identified as a prognostic predictor (*P* > 0.05) (Fig. 2). This could be due to the small sample size and the rarity of the cohort, which limits the statistical power of such comparison .

## Discussion

HCC-PM is generally regarded as an advanced stage of disease with a poor prognosis, often managed with palliative chemotherapy or targeted therapy [16]. Lung metastasis is the most frequently encountered site, accounting for 55.0% of cases, while peritoneal metastasis is rare [13, 21, 22]. The etiology of HCC-PM remains elusive, yet several prominent risk factors have been identified that contribute to its occurrence. These factors encompass the rupture of the primary hepatocellular carcinoma, the potential for tumor cell implantation during surgical resection, invasive diagnostic procedures such as liver biopsy and radiofrequency ablation, as well as the presence of poorly differentiated histological subtypes [3, 23].

Current guidelines by the American National Comprehensive Cancer Network suggest sorafenib, supportive care, or clinical trials for HCC-PM. A multicenter, placebo-controlled, double-blind study by Josep M et al. investigated the use of sorafenib in treating unresectable or metastatic hepatocellular carcinoma, reporting a modest median survival of merely 10.7 months [24]. Congruent with these findings, an Asian phase III trial demonstrated that sorafenib therapy for metastatic hepatocellular carcinoma resulted in a similarly limited median survival of 6.5 months [12]. In contrast, regorafenib has emerged as a viable alternative for the management of advanced hepatocellular carcinoma. Jordi Bruix and colleagues conducted a multicenter, placebo-controlled double-blind study that showed regorafenib to be associated with a median survival of 10.6 months in patients with advanced disease [25]. Notably, both sorafenib and regorafenib have been found to outperform placebo in terms of patient survival. The median OS ranges from 6.5 to 16.4 months when treated with targeted agents or immunotherapy [24–28]. Furthermore, a phase III clinical study suggested that the FOLFOX4 regimen may extend OS, PFS, and response rate (RR) compared to doxorubicin monotherapy in the setting of advanced hepatocellular carcinoma [29]. Despite these therapeutic options, multiple studies have indicated that non-surgical treatments do not significantly prolong patient survival [24, 29, 30]. Interestingly, HCC-PM was not identified as an independent prognostic factor for advanced hepatocellular carcinoma [31]. Additionally, there is evidence to suggest that resection of extrahepatic metastases could confer a survival benefit for selected patients with hepatocellular carcinoma [15].

Over the past few decades, the integrated treatment strategy centered on CRS + HIPEC has revolutionized the management of various peritoneal cancers. This approach has not only become the recommended protocol for primary and secondary peritoneal malignancies but also the standard of care for the peritoneal metastasis of ovarian cancer, as corroborated by numerous studies [9, 32–36]. Evidence from several investigations suggests that a subset of patients with isolated extrahepatic metastasis may experience significant benefits from surgical excision [4, 12, 14, 21]. For example, Yeh et al. reported in a 2004 study that the 1-year, 3-year, and 5-year survival rates for patients with HCC-PM were 62.5%, 34.1%, and 30.1%, respectively, following surgical removal of peritoneal deposits [11]. Additionally, in cases of metachronous HCC-PM, surgical intervention was associated with a median OS of 12.5 months, in stark contrast to the 2.1 months observed with non-surgical treatments [13]. Patients with resectable disease exhibited a markedly improved median OS of 33.0 months, compared to just 14.0 months for those with unresectable disease [37]. Furthermore, a lower PCI score was conducive to achieving complete cytoreduction (CC0 ~ 1), translating into an extended survival with a median OS of 35.6 months. However, attaining complete resection proved challenging in cases with high PCI scores, and these patients were more prone to complications [14]. Furthermore, An international multicentric cohort study conducted by the Peritoneal Surface Oncology Group International (PSOGI) has further substantiated the safety and efficacy of CRS + HIPEC for selected patients with HCC-PM. This study reported a median OS of 46.7 months and a 5-year recurrence-free survival rate of 37.0%, despite nearly half of the patients experiencing SAEs [16]. In another study, optimal outcomes were also observed for HCC-PM, with a median OS of 15.7 months and 1-year, 2-year, and 4-year survival rates of 66.7%, 33.3%, and 33.3%, respectively, even in patients with a higher PCI than previous investigations. However, it is noteworthy that the 3-year recurrence rate reached 100% [17].

A growing body of evidence has documented favorable outcomes in the management of HCC-PM, with some patients experiencing prolonged PFS [4, 10, 12, 15, 21, 30]. Median PCI scores from previous investigations have varied, ranging from 7 to 18.5 [16, 17, 38]. In contrast, our study reported a higher median PCI of 25, which suggests that achieving satisfactory cytoreduction is more demanding. Despite the increased difficulty, half of the patients in our study achieved complete or near-complete cytoreduction (CC0 ~ 1). The majority of patients received postoperative treatment, with several undergoing multiple treatments. Remarkably, our results were superior to those of earlier studies [12, 14, 17, 38, 39], underscoring the potential for improved prognosis with refined therapeutic strategies. These findings highlight the importance of individualized treatment plans and the potential for improved outcomes even in cases with a higher PCI. They also emphasize the value of meticulous surgical techniques and the role of adjuvant therapies in enhancing patient survival. Further research is necessary to continue refining treatment protocols and to identify factors that may contribute to successful outcomes in patients with HCC-PM.

## Conclusions

The application of CRS + HIPEC offers significant benefits to patients with HCC-PM. A selected group of patients may achieve prolonged PFS. Incorporating CRS + HIPEC into the treatment paradigm can thus be considered a strategic therapeutic option for patients with HCC-PM.

## Data Availability

The datasets analyzed during the current study are available from the corresponding author upon reasonable request.
